# Feasibility randomized controlled trial of a home-based support program for family caregivers of people with Alzheimer’s disease

**DOI:** 10.3389/fnagi.2026.1744279

**Published:** 2026-01-27

**Authors:** Hongli Liu, Yuanli Jiang, Jiajia Chen, Wenqing Pan, Ping Ju, Ling Li, Li Zhang, Yunxing Cao, YuHang Zhu

**Affiliations:** 1 Department of Critical Care Medicine, Chongqing General Hospital, Chongqing University, Chongqing, China; 2 Nursing School of Zunyi Medical University, Zunyi, Guizhou, China; 3 Department of Geriatrics, Affiliated Hospital of Zunyi Medical University, Zunyi, Guizhou, China; 4 Early Clinical Research Ward, Affiliated Hospital of Zunyi Medical University, Zunyi, Guizhou, China; 5 Discipline Inspection and Supervision Office of Affiliated Hospital of Zunyi Medical University, Zunyi, Guizhou, China; 6 Key Laboratory of Brain Function and Brain Disease Prevention and Treatment of Guizhou Province, Affiliated Hospital of Zunyi Medical University, Zunyi, Guizhou, China

**Keywords:** Alzheimer’s disease, family caregivers, health-related quality of life, home care, randomized controlled trial

## Abstract

**Objective:**

This study aimed to develop and preliminarily evaluate the feasibility of a home-based care program for family caregivers of individuals with Alzheimer’s disease (AD).

**Methods:**

We developed a home-based intervention for AD caregivers through systematic literature review and two-round Delphi consensus (18 experts; authority coefficients 0.88–0.91). This feasibility randomized controlled trial enrolled 61 primary caregivers and assigned them to either an experimental group (*n=31*) receiving a structured, evidence-based, 3-month home-care protocol, or a control group (*n=30*) receiving conventional nursing guidance.

**Results:**

The Delphi process achieved strong expert consensus. Post-intervention, caregivers in the intervention group demonstrated significant improvements in AD knowledge scores, anxiety reduction, and psychological domain QOL-AD scores compared to baseline (*P* < 0.05). While total QOL-AD scores increased in the intervention group, between-group differences were not statistically significant.

**Conclusion:**

The home-based care program proved feasible and effective in enhancing AD caregiver knowledge and mental health outcomes. These promising findings support the need for larger-scale efficacy trials to further validate clinical utility.

## 1 Introduction

Alzheimer’s disease, a neurodegenerative disorder caused by damage to nerve cells or neurons, is the most common type of dementia, accounting for 60–80% of dementia cases (2024). It is primarily characterized by progressive cognitive dysfunction, including memory, attention, comprehension, and language impairments, as well as mental and behavioral abnormalities. By 2050, the global number of dementia patients is projected to exceed 100 million. China currently has the largest population of AD and other dementia patients, comprising approximately 29.8% of the global total, with one of the fastest growth rates in the world (Wang G. et al., 2024). Studies have found that among people aged 65 years and older, the survival time of AD after diagnosis is about 4–10 years, and the survival time of some patients can be extended to 20 years (Liang et al., 2021). Due to the progressive decline in cognitive function and self-care ability among patients, the day-to-day care for individuals with dementia is mainly provided by family members or specialized long-term care facilities. In the United States, when the care needs of individuals with dementia exceed the capacity of their families, they typically transition to long-term care facilities for professional support. However, due to the underdeveloped nursing home system and the enduring influence of traditional family values, over 90% of dementia patients in China primarily rely on family caregivers (Yang et al., 2020). The prolonged implementation of China’s one-child policy has reduced family size, whether family members sacrifice employment opportunities and endure income loss to take on caregiving responsibilities, or opt to hire professional caregivers or enroll patients in nursing homes and care facilities for expert attention, these decisions invariably lead to significant economic burdens. In 2023, informal caregivers of people with AD or other dementia provided nearly 18.4 billion hours of unpaid care, valued at a total of $346.6 billion (2024). China’s dementia strategy clearly highlights that family caregivers are indispensable and valuable assets.

AD constitutes an escalating public-health priority. Yet early-stage symptoms are often overlooked until substantial cognitive decline has occurred. Delayed diagnosis of Alzheimer’s disease is associated with helplessness and powerlessness among family members (Cox et al., 2025). Furthermore, AD is a stigmatized condition that significantly impacts both diagnosed individuals and their caregivers (Rosin et al., 2020). When confronting the difficulties and pressures of patients, family caregivers often hesitate to discuss the patient’s condition. Among them, most believe such discussions are unlikely to yield meaningful changes beyond emotional comfort. This perception highlights significant deficiencies in AD care resources and support systems. Kojima et al. (2024) found that AD caregivers score lower across all health-related quality-of-life domains, especially in mental health and social functioning. Across multiple studies (Matthews et al., 2019; Livingston et al., 2020; Mattos et al., 2024), caregivers of individuals with AD consistently report poorer self-rated physical health, a higher prevalence of chronic conditions, and elevated all-cause mortality; they also exhibit high levels of psychological distress, including anxiety, despair, and social isolation. Research shows that 59% of family caregivers of individuals with dementia experience high or very high levels of psychological stress during the caregiving process (2024). Such anxiety among AD caregivers often stems from constant vigilance over patient safety (such as wandering and falling), anticipatory grief over the irreversible deterioration of the condition, and profound doubts about their long-term competence in the caregiving role. Mastery of knowledge related to AD is a fundamental cognitive resource for family caregivers to cope effectively with care challenges. The behavioral and psychological symptoms of AD are complex and variable; if caregivers lack an accurate understanding of the pathological roots, they are likely to misinterpret a symptom as the patient’s intentional resistance. This not only exacerbates communication difficulties but may also lead caregivers to adopt ineffective or even harmful coping strategies. Due to a lack of systematic care knowledge and skills, family caregivers often experience helplessness and significant anxiety. Moreover, many undertake this role without sufficient information or external support from the outset. Even today, as awareness about Alzheimer’s disease grows, they frequently seek help only in emergencies. Excessive psychological distress and a lack of caregiving knowledge can weaken caregivers’ emotional resilience and diminish their sense of competence. This may result in burnout and, in severe cases, neglect or abuse, which can seriously impact the patients’ quality of life.

Globally, supportive interventions for family caregivers are crucial strategies in dementia care. These interventions aim to reduce caregivers’ burden and improve patients’ quality of life-an approach widely advocated by the World Health Organization and Alzheimer’s Disease International that aligns with person-centered care. Therefore, this study aims to develop and validate a home care program for AD caregivers, which is reported as follows.

## 2 Construction of home care program for family caregivers of AD

### 2.1 To formulate a home care plan for family caregivers of AD

#### 2.1.1 Data sources and search strategy

Following the “6S” model of evidence-based resources, we systematically searched relevant databases and websites, including: BMJ Best Practice, UpToDate, the National Guideline Clearinghouse (NGC), the U.K. National Institute for Health and Care Excellence (NICE) guidelines network, Medical Pulse, Australia, the Joanna Briggs Institute (JBI) Center for Evidence-based Health Care database, The Cochrane Library, CINAHL, MEDLINE, Web of Science (WoS), EMbase, PubMed/Medline, OVID, China National Knowledge Infrastructure (CNKI), China Biology Medicine Literature, Wanfang, and VIP database. The search timeframe spanned from January 1, 2000, to December 31, 2023. English keywords included: “Alzheimer’s Diseases/Dementia Cognitive Disorders/Neurocognitive Dis- orders,” “Family Caregiver/Informal Caregiver/Spouse Caregiver,” “Family Nursing/Family Care/Nursing Skills/Nursing Methods,” “Guidelines/Best Practices/Expert Consensus/Evidence Summary/ Systematic Review/Meta-analysis/Randomized Controlled Trials/ Original Study.”

#### 2.1.2 Eligibility criteria

Inclusion criteria included the following: (1) Family caregivers of people with Alzheimer’s disease are the study population; (2) Studies focus on home-based care; (3) Article types: guidelines, systematic reviews, best-practice statements, expert consensus documents, meta-analyzes, evidence summaries, or randomized controlled trials; (4) Publications written in English or Chinese; (5) Most recent version or update. Literature exclusion criteria included: (1) Articles rated as low quality after methodological appraisal. (2) Full text unavailable. (3) Duplicate publications or translations. (4) Systematic reviews still in the protocol phase or already incorporated into included guidelines.

#### 2.1.3 Quality assessment

The 2017 edition of the JBI critical appraisal tool was employed to rate methodological quality and ensure robust evidence for clinical decision-making. Two well-trained researchers reviewers independently performed the quality assessment and data extraction; disagreements were resolved by a third expert experienced in the field. Extracted data were cross-checked and iteratively refined until consensus was achieved.

#### 2.1.4 The results of the literature review

A total of 1,973 articles were retrieved. After deduplication using software and exclusion of literature that did not meet the inclusion criteria, 13 articles were retained. These included 4 guidelines, 6 expert consensus documents, 2 systematic reviews, and 1 evidence summary. Following the evidence synthesis, a preliminary intervention plan was developed.

### 2.2 Delphi expert consultation

Using the Delphi method, 18 Alzheimer’s disease experts participated in a two-round written survey. Response rates were 90% (Round 1) and 100% (Round 2), with disagreement rates decreasing from 50 to 11.11%. Authority coefficients (reflecting expert credibility) were 0.88 and 0.91, respectively. Experts provided positive feedback: Average item scores improved from 4.11 to 5.00 (Round 1) to 4.28–5.00 (Round 2). Coefficients of variation for both rounds were below 0.25, demonstrating strong consensus.

## 3 Application of home care program for family caregivers of Alzheimer’s disease

### 3.1 Study subjects

Using the purposive sampling method, family caregivers from the Department of Neurology at the Affiliated Hospital of Zunyi Medical University, who met the inclusion and exclusion criteria between January and November 2024, were selected as study participants. This study was approved by the Ethics Committee of the Affiliated Hospital of Zunyi Medical University (Approval No. KLL-2022-460) and registered with the China Clinical Trial Center (No. ChiCTR2200060485).

#### 3.1.1 Eligibility and attrition criteria

##### 3.1.1.1 Inclusion criteriam

(1) Age of 45 years and above. According to one study (Dahlberg et al., 2007), informal caregivers are most common among individuals aged 45 years and older. Therefore, this study selected individuals aged 45 years and older within the survey scope as the target population. (2) Having primary care responsibility for elderly with Alzheimer’s disease, and living with them for at least 3 months. (3) All patients were clinically diagnosed at the same tertiary hospital, the diagnosis of Alzheimer’s disease was strictly based on the diagnostic criteria outlined in the Chinese Guideline for the Diagnosis and Treatment of Dementia and Cognitive Disorders (Tian et al., 2021). The diagnosis was made by an attending neurologist specializing in cognitive disorders within the hospital’s department. (4) Voluntary participation with signed informed consent.

##### 3.1.1.2 Exclusion criteria

(1) Presence of serious diseases in vital organs (e.g., heart, brain, lung, or kidney). (2) Diagnosis of mental disorders. (3) Severe hearing, speech, comprehension, or cognitive impairment. (4) Employment as a paid caregiver (e.g., nursing assistants, nannies).

##### 3.1.1.3 Elimination criteria

(1) Change of primary family caregiver during the intervention period. (2) Transfer of AD patients to long-term care facilities during the intervention period. (3) Voluntary withdrawal from the study during the intervention period. (4) Death of AD patients or family caregivers due to illness or accident. (5) Loss of contact with participants during the intervention period.

##### 3.1.1.4 Drop-out criteria

(1) Voluntary withdrawal from the study during the intervention period. (2) Death of AD patients or family caregivers due to illness or accident during the intervention period. (3) Participants lost to contact during the intervention period.

### 3.2 Sample size determination

Taking health-related quality of life as the outcome indicator, this study assumed that the sample size of the two groups was equal, and the sample size estimation formula according to the comparison of the mean of the two samples was as follows (Huang, 2015):$\mathrm{N1}=\mathrm{N2}=2\,{\left[\frac{\left(Z_{\alpha /2}+Z_{\beta }\right)\,\sigma }{\delta }\right]}^{2}$, N1 and N2 were the required sample size for each group; Using two-tailed test, α = 0.05, β = 0.10, Z_α /2_ = Z_0.05/2_ = 1.960, Z_β_ = Z_0.20_ = 0.842. The values of σ and δ were calculated by referring to relevant literature (Ma et al., 2023). Calculate the required sample size for each group to be 32 cases, considering the loss of follow-up rate of about 15%, N = N*1/(1–15%) determined the sample size for inclusion was 74.

### 3.3 Research methods

#### 3.3.1 Research tools

##### 3.3.1.1 Assessment tools for family caregivers

(1) Case report form (CRF) : including basic and medical information. Basic information such as name, gender, age, etc., and medical information mainly collected concomitant diseases of various systems.

(1) SF-36 scale: The Chinese SF-36, translated by Li et al. (2002), demonstrated acceptable reliability (Cronbach’s α: 0.72–0.88) across six domains, except social functioning and vitality. This 36-item questionnaire evaluates eight health domains (physical functioning, bodily pain, vitality, role-physical, role-emotional, general health, social functioning, mental health) and generates standardized Physical Component Summary (PCS), Mental Component Summary (MCS), and Total scores via proprietary algorithms. Widely validated in diverse populations, including dementia caregivers (Qi et al., 2021; Yang et al., 2022), it serves as a robust measure of health-related quality of life in epidemiological surveys, policy assessments, and clinical trials.

(1) Chinese version of Alzheimer’s Disease Knowledge Scale (ADKS): It contains 30 items, which aims to accurately assess the knowledge level of AD patients, caregivers and medical professionals (Carpenter et al., 2009). The Chinese version of ADKS was translated by Chinese scholars and strictly tested for reliability and validity. In terms of test-retest reliability, the score of the scale ranged from 0.732 to 0.879, indicating good time stability. In addition, Cronbach’s α coefficient was 0.756, which further confirmed the internal consistency of the scale (He et al., 2013). ADKS is the most commonly used assessment tool for AD knowledge at home and abroad, which has high reliability, good validity and adaptability.

(1) Anxiety assessment of family care: Zung Self-rating Anxiety Scale (SAS) developed by Professor Zung in 1971 was used for evaluation (Zung, 1971). Wang X. et al. (2024) tested the reliability and validity of SAS among community residents in China and found that Cronbach’s α coefficient of the scale was 0.733, indicating that it had good internal consistency. Although the SAS scale was developed earlier, both of them are convenient, quick and easy to master, so they continue to be widely used in the evaluation of anxiety in medical research (Sun et al., 2022).

##### 3.3.1.2 Assessment tools for patients

(1) General information questionnaire of patients, including name, gender, age, etc.

(2) Assessment of cognitive function: The Montreal Cognitive Assessment (MoCA) is one of the most widely utilized tools for evaluating cognitive function in clinical settings, the MoCA was developed and validated by Nasreddine et al. (2005) in Canada based on clinical experience and with reference to the cognitive items and scoring of the MMSE (Mini-Mental State Examination). Initially cross-culturally adapted for Chinese populations by Wang and Wang (2007), the scale was formally implemented in China’s clinical practice that year. The Chinese version of MoCA is adapted for elderly individuals with low education levels and demonstrates no ceiling effect in highly educated elders. The MoCA has a total score of 30 points, and a score less than 26 indicates impaired cognitive function.

(3) The Clinical Dementia Rating scale (CDR) is a globally used staging instrument for assessing dementia severity (Homma et al., 2006). The scoring criteria are as follows: 0 points: Normal cognitive function; 0.5 points: Very mild dementia (or suspected dementia); 1 point: Mild dementia; 2 points: Moderate dementia; 3 points: Severe dementia. This scale demonstrates high reliability, strong internal consistency, and good responsiveness. Its design minimizes confounding effects from age, sex, education/literacy level, and racial/ethnic or cultural factors (Yang et al., 2021).

(1) Alzheimer’s Quality of Life scale (QOL-AD): The QOL-AD was designed by Logsdon et al to evaluate the quality (Logsdon et al., 2002) of life of dementia patients. Zhang et al. (2013) translated and revised the QOL-AD into Chinese. The Cronbach’s α coefficient of the Chinese revised QOL-AD (caregiver version) was 0.869, with good internal consistency. In addition, the scale of the total weight measuring reliability is 0.835, shows goodtime stability and consistency.

#### 3.3.2 Assessment tools for caregiver

(1) Main outcome indicators: SF-36 points.

(2) The secondary outcome indicators: Alzheimer’s disease knowledge scale score, anxiety self-assessment scale score, and Alzheimer’s disease quality of life scale (caregivers) score.

#### 3.3.3 Intervention methods

##### 3.3.3.1 Research team

The research team included one master’s tutor (Neurology Department), one head nurse in the department of neurology, one neurologist, one rehabilitation doctor, two nurses in the department of neurology, the researcher himself, and three postgraduate students. The specific division of labor is shown in Table 1.

**TABLE 1:** Specific allocation of responsibilities among research team members.

Team member	Specific work
Master’s supervisor	Primarily responsible for project design, quality assurance, and facilitating inter-departmental coordination and communication.
Head nurse	Primarily responsible for coordinating with research participants and overseeing project implementation.
Neurologist	Primarily responsible for diagnosing Alzheimer’s disease and for overseeing the development and implementation of the intervention plan.
Rehabilitation physician	Primarily responsible for overseeing and guiding the development and implementation of the intervention plan.
Neurology nurse 1	Primarily responsible for implementing the intervention program on the 10th-floor neurology ward during participants’ hospital admissions.
Neurology nurse 2	Primarily responsible for implementing the intervention program on the 11th-floor neurology ward during participants’ hospital admissions.
Graduate student 1	Administered the standard nursing protocol to the control group during home care visits.
Graduate student 2	Implemented the study-specific intervention program for the intervention group during home care services.
Graduate student 3	Primarily responsible for the systematic collection, organization, and management of research data throughout the study.
Researcher herself	Responsible for overseeing the overall program design, coordinating team member training, conducting statistical analyses, contributing to manuscript preparation, and coordinating the implementation process.

##### 3.3.3.2 Training of research team members

In 1 month before the intervention to the special training of project participants, the researchers explain how application solutions, including plan, implementation process and the specific content of the questionnaire method of use, etc., using multimedia teaching a total of 3 times, before formally to carry out the task completed one simulation practice examination, is not eligible for training until the examination qualified again, until the consistency of the collection standard is reached.

##### 3.3.3.3 The duration of intervention

Studies (Balvert et al., 2024) have found that interventions for family caregivers of AD patients lasting 3–4 months are generally more effective than shorter or longer ones. Considering the time and workload constraints of the research project, the research team decided upon a 3-month intervention duration for this study. Designating the 3-month assessment as the primary endpoint provides a critical window for evaluating the sustainability of the intervention, while allowing adequate time for caregivers to acquire and consolidate new care skills and coping strategies (e.g., mindfulness exercises (Kor et al., 2019), app-based information management (Ruggiano et al., 2024), telehealth technology to link caregivers with dementia care experts for in-home caregiving support (Williams et al., 2018)). This 12-week interval is also operationally advantageous: it is sufficiently long to detect clinically meaningful change, yet short enough to contain study costs and minimize participant attrition, thereby furnishing the preliminary evidence required for a definitive, large-scale randomized controlled trial.

##### 3.3.3.4 Grouping method

Patients were divided into intervention and control groups through stratified randomization. Family caregivers who met the inclusion and exclusion criteria were stratified based on their patients’ Alzheimer’s disease severity (mild, moderate, or severe). Within each stratum, they were randomly assigned to the intervention or control group using a computer-generated random sequence to ensure baseline comparability. The Department of Neurology occupied two floors. The intervention group was assigned to the 10th floor, while the control group was assigned to the 11th floor.

##### 3.3.3.5 Form of intervention

**FIGURE 1:**
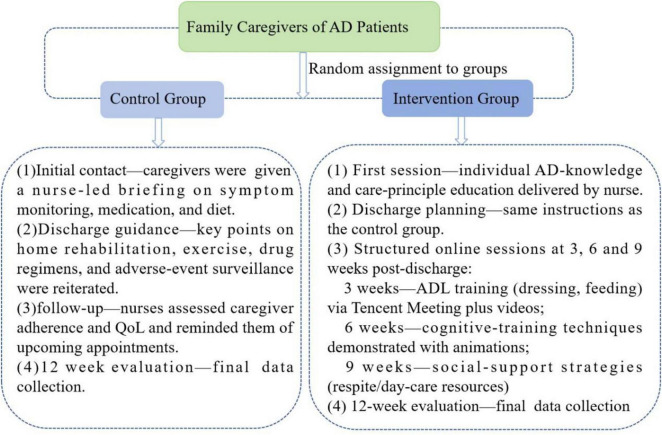
Interventions for the two groups of AD family caregivers.

Within the hospital setting, primary family caregivers were given standardized guidance on securing informed consent. For participating patients diagnosed with Alzheimer’s disease and their family caregivers, the assessment instruments were subsequently administered, and corresponding records were created. A schematic of the interventions delivered to the two distinct groups of AD family caregivers is presented in Figure 1.

*Control group:* The conventional nursing guidance model was implemented as follows: (1) Initial Contact: The researcher and the primary family caregiver exchanged WeChat contacts to establish rapport. A standardized oral explanation was provided, covering symptom monitoring, dietary guidance, and medication management. (2) Discharge: The responsible nurse conducted face-to-face education, reinforcing guidance on home-based diet, rehabilitation exercises, medication adherence, drug-effect monitoring, and the need for immediate medical consultation in case of discomfort. (3) One-month Follow-up: A telephone follow-up was conducted by the outpatient or responsible nurse using a standardized script to assess caregiving status and quality of life, and to remind the patient of scheduled reviews. (4) Three-month Follow-up: A face-to-face home visit was performed to collect data from the primary family caregiver. During these contacts, primary family caregivers were also provided with information on the clinical presentation, pharmacotherapy, and prevention strategies related to Alzheimer’s disease.

*Intervention group*: The intervention was delivered according to the evidence-based home-care protocol developed for family caregivers of individuals with AD: (1) Initial Intervention: The researcher and the primary family caregiver exchanged WeChat contacts to establish rapport. The responsible nurse provided a one-to-one explanation of AD-related knowledge, constituting the first component of the protocol. (2) Discharge: The responsible nurse supplemented the initial education with additional discharge guidance, reinforcing topics such as home diet, rehabilitation exercises, medication adherence, drug-effect monitoring, and instructions for seeking immediate medical consultation. (3) Resource Development: Animated videos were produced and peer-reviewed for scientific accuracy. The videos covered medication knowledge, care for basic and instrumental activities of daily living, home-based cognitive training, management of neuropsychiatric symptoms, respite services, and an introduction to day-care centers. This content was also converted into PowerPoint presentations for delivery via Tencent Meeting. (4) Ongoing Sessions: Every three weeks post-discharge, online Tencent Meetings were held to implement the intervention protocol. The first session covered techniques for assisting with dressing and eating, demonstrated via animated videos (Part 2 of the protocol). The second session addressed cognitive training techniques (Part 3). The third session introduced respite services and day-care centers (Part 4). (5) Three-month Follow-up: A face-to-face visit was conducted to collect data from the primary caregiver. Caregivers were contacted in advance; if unable to attend, they received a post-session telephone call to relay the content, and the instructional videos were provided for self-study.

#### 3.3.4 Blinding

Family caregivers and the graduate students conducting clinical assessments were blinded to whether families received the study protocol or conventional care. During hospitalization, intervention methods employed by neurology nurses on the 10th and 11th floors operated independently to prevent cross-contamination. Home care measures were implemented by separate researchers, the intervention group used the intervention program of this study, and the control group used the routine nursing program. After every intervention, family caregivers were asked how they felt about the intervention to confirm that they did not know which intervention they received. Post-intervention, data were collected from both caregiver groups by an independent researcher (Graduate student 3).

#### 3.3.5 Statistical methods

Two graduate students double-checked the data in an Excel spreadsheet before importing it into SPSS 29.0 for statistical analysis. Baseline data (age, BMI) were described using descriptive statistics for continuous variables. Data meeting independence, normality, and homogeneity of variance assumptions were analyzed via independent-sample t-tests. Categorical variables (e.g., gender, occupation) were summarized as frequencies and percentages, with group comparisons using Chi-square or Fisher’s exact tests. All tests were two-tailed, with *P* < 0.05 considered statistically significant.

#### 3.3.6 Quality control

##### 3.3.6.1 The preparation stage

To ensure that the intervention is scientific, feasible and applicable, the team first systematically searched, screened and evaluated the literature to draft the initial protocol. Relevant experts were then invited to review and refine the protocol. Finally, the research staff were trained before implementation to guarantee consistency in both the delivery of the intervention and the collection of data. To minimize bias, all patients were diagnosed at a single tertiary hospital, and CDR ratings were obtained independently of the initial diagnostic work-up by the neurologist using the standard protocol based on separate interviews with patients and their primary caregivers.

##### 3.3.6.2 Implementation stage

We fully informed all patients and family caregivers of the study’s purpose and significance. To enhance participation: (1) free MRI scans were provided to the patients and family caregivers; (2) remote booster sessions were delivered via WeChat every three weeks; and (3) flexible scheduling (evening/weekend slots) and optional individual or group formats were offered. All intervention-group training was conducted exclusively by the research team, while data collection was performed by third-year graduate students who had no involvement in the intervention, ensuring that a blinded approach was maintained throughout.

## 4 Results

### 4.1 sample attrition rates

**FIGURE 2:**
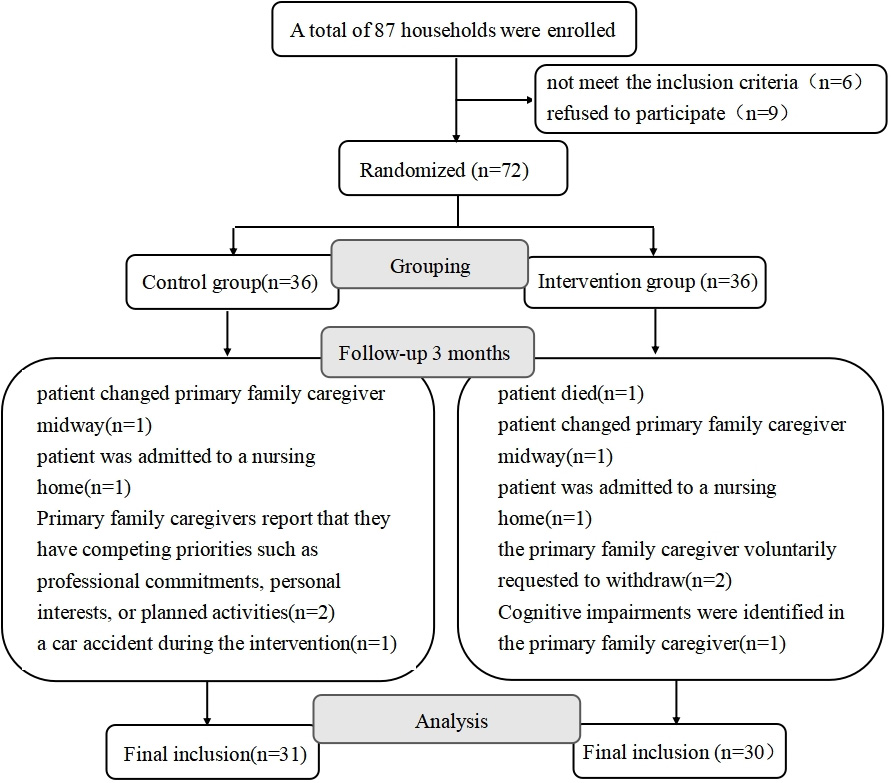
Flowchart for participant recruitment.

A total of 87 groups of AD patient-caregiver families were recruited. After excluding 6 cases who did not meet the criteria and 9 cases who refused to participate, 72 cases were finally included. During the intervention period, 11 participants were lost to follow-up, resulting in a total attrition rate of 15.28%. The primary reasons for withdrawal in both the intervention and control groups were health-related issues: one caregiver was involved in a motor vehicle accident, one caregiver developed cognitive impairment, two patients were transferred to nursing homes, and one participant died during the study. Another significant reason was the perceived stress among family caregivers: two families changed their primary caregivers; two caregivers cited other priorities (e.g., professional commitments, personal interests or planned activates); and two requested voluntary withdrawal. Figure 2 details the participant recruitment and retention process from baseline through follow-up assessments.

### 4.2 Comparison of general data

#### 4.2.1 Comparison of general conditions between the two groups

There were no statistically significant differences in demographic and clinical characteristics, including gender, age, place of residence, and education level, between the intervention group and the control group (*P* > 0.05). The proportion of female patients was significantly higher at 68.85%. The educational attainment of the patients was generally low, with 49.18% (*n* = 30) having received less than 1 year of formal education. There were no statistically significant differences between the two groups in terms of other chronic diseases, MoCA scores, and disease severity (*P* > 0.05). The overall MoCA score was relatively low (7.26 ± 4.26), and according to the Clinical Dementia Rating scale, the majority of participants were classified as moderate-stage dementia (CDR = 2, *n* = 26, 46.62%). For detailed information, please refer to Table 2.

**TABLE 2:** Comparison of the general data of the two groups of AD patients.

Variable	Categories	Total (*n* = 61)	Control group (*n* = 31)	Intervention group (*n* = 30)	Test statistic	*P*
Gender					0.036	0.849^a^
	Male	19 (31.15)	10 (32.26)	9 (30.00)		
	Female	42(68.85)	21(67.74)	21 (70.00)		
Age		75.49 ± 6.81	75.74 ± 7.12	75.23 ± 6.58	0.290	0.773^b^
BMI		23.23 ± 3.91	22.80 ± 3.71	23.69 ± 4.12	−0.886	0.378^b^
Residential location					0.435	0.510^a^
	Rural	34 (55.74)	16 (51.61)	18 (60.00)	
	Town	27 (44.26)	15 (48.39)	12 (40.00)	
Education (year)					2.445	0.495^c^
	≤ 1	30(49.18)	17 (54.84)	13 (43.33)		
	2 ∼ 6	10 (16.39)	4 (12.90)	6 (20.00)		
	7∼9	14 (22.95)	8 (25.81)	6 (20.00)		
	> 9	7 (11.48)	2 (6.45)	5 (16.67)		
Having other types of chronic diseases						1.000^c^
b	0	15 (24.59)	8 (25.81)	7 (23.33)		
	1 ∼2	37 (60.66)	19 (61.29)	18 (60.00)		
	≥ 3	9 (14.75)	4 (12.90)	5 (16.67)		
MoCA		7.26 ± 4.26	6.81 ± 4.24	7.73 ± 4.31	−0.847	0.400^b^
Degree of severity					0.042	0.979^a^
	Mild	18 (29.51)	9 (29.03)	9 (30.00)		
	Moderate	26 (42.62)	13 (41.94)	13 (43.33)		
	^Heavy^	17 (27.87)	9 (29.03)	8 (26.67)		

^a^For chi-square test *P-*values. ^b^For *t-*test *P-*values. ^c^*P-*values for Fisher accurate inspection.

#### 4.2.2 The general data of two groups of family caregivers

There was no significant difference in gender, age, residence and other general demographic data of caregivers between the two groups (*P* > 0.05), as shown in Table 3. Overall men (54.10%), the more and more as the spouse relationship (63.93%), the vast majority of family caregivers are not working or retired, level of education for elementary school or junior high school, 31 family caregivers with 1–2 kinds of chronic disease, with hypertension and diabetes are the most common.

**TABLE 3:** Two groups of family caregivers general data information.

Variable	Categories	Total (*n* = 61)	Control group (*n* = 31)	Intervention group (*n* = 30)	Test statistic	*P*
Gender					0.828	0.363^a^
	Male	33 (54.10)	15 (48.39)	18 (60.00)		
	Female	28 (45.90)	16 (51.61)	12 (40.00)		
Age		66.21 ± 11.94	66.03 ± 12.04	66.40 ± 12.04	−0.119	0.905^b^
BMI		23.76 ± 3.17	23.77 ± 3.25	23.75 ± 3.14	0.014	0.989^b^
Ethnicity						0.671^c^
	Han nationality	56 (91.80)	29 (93.55)	27 (90.00)		
	Ethnic minorities	5 (8.20)	2 (6.45)	3 (10.00)		
Residential location					0.021	0.886^a^
	Rural	34 (55.74)	17 (54.84)	17 (56.67)		
	Towns	27 (44.26)	14 (45.16)	13 (43.33)		
Marital status						1.000^c^
Have a spouse		59 (96.72)	30 (96.77)	29 (96.67)		
	Single	2 (3.28)	1 (3.23)	1 (3.33)		
Relationship					0.009	0.923^a^
	Spouse	39 (63.93)	20 (64.52)	19 (63.33)		
	Children	22 (36.07)	11 (35.48)	11 (36.67)		
The status of current job						1.000^c^
	None/retired	56 (91.80)	28 (90.32)	28 (93.33)		
	on-the-job	5 (8.20)	3 (9.68)	2 (6.67)		
Education (year)						0.466^c^
	≤1	7 (11.48)	3 (9.68)	4 (13.33)		
	2∼6	23 (37.70)	13 (41.94)	10 (33.33)		
	7∼9	21 (34.43)	12 (38.71)	9 (30.00)		
	> 9	10(16.39)	3 (9.68)	7 (23.33)		
The type of chronic diseases						1.000^c^
	0	21 (34.40)	11 (35.48)	10 (33.33)		

^a^For chi-square test *P-*values. ^b^For *t-*test *P-*values. ^c^*P-*values for Fisher accurate inspection.

### 4.3 Comparative analysis of baseline conditions for each index between the two groups prior to intervention

**FIGURE 3:**
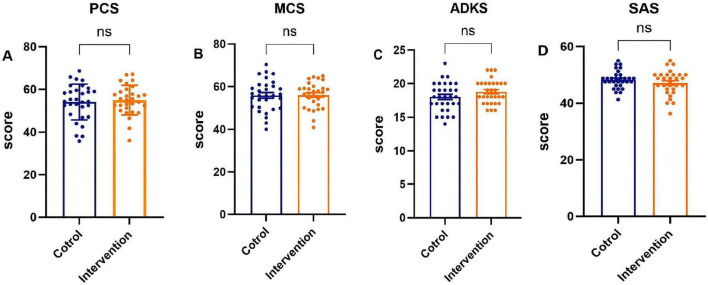
Baseline scores on various scales for the two groups of family caregivers. **(A)** The Physical Component Summary (PCS) score, **(B)** the Mental Component Summary (MCS) score, **(C)** the Chinese version of Alzheimer’s Disease Knowledge Scale (ADKS) score, **(D)** the Self-rating Anxiety Scale (SAS) score.

The scores of the PCS and MCS of family caregivers in the two groups, Alzheimer’s disease knowledge score, anxiety self-assessment scale score before the intervention had no statistical significance (*P >* 0.05), as shown in Figure 3.

### 4.4 The indices of the two groups of family caregivers were compared both at baseline and post-intervention

#### 4.4.1 Scores of each index of family caregivers in the two groups before and after intervention

**FIGURE 4:**
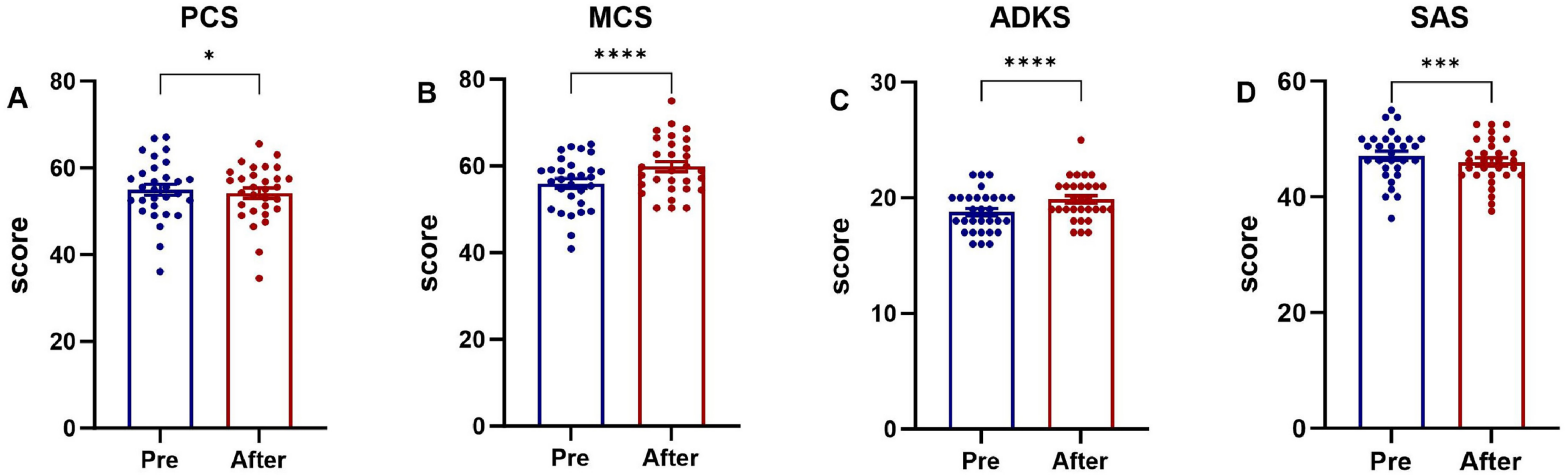
Comparison of pre-intervention and post-intervention scores in family caregivers (intervention group). **(A)** The Physical Component Summary (PCS) score, **(B)** the Mental Component Summary (MCS) score, **(C)** the Chinese version of Alzheimer’s Disease Knowledge Scale (ADKS) score, **(D)** the Self-rating Anxiety Scale (SAS) score. *P < 0.05, ***P < 0.001, ****P < 0.0001.

**FIGURE 5:**
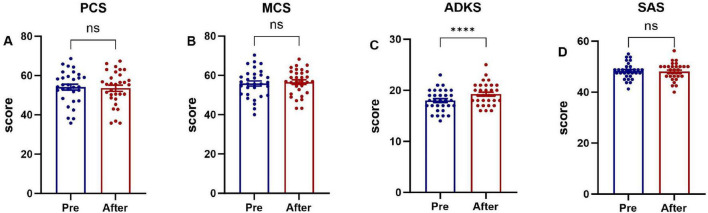
Comparison of pre-intervention and post-intervention scores in family caregivers (control group). **(A)** The Physical Component Summary (PCS) score, **(B)** the Mental Component Summary (MCS) score, **(C)** the Chinese version of Alzheimer’s Disease Knowledge Scale (ADKS) score, **(D)** the Self-rating Anxiety Scale (SAS) score. ****P < 0.0001

Family caregivers in the intervention group who received the evidence-based home-care protocol showed significant improvements (*P* < 0.05) in physical health, mental health, ADKS, and SAS scores post-intervention, as shown in Figure 4. Family caregivers in the control group were provided with the conventional nursing guidance model, only the ADKS score improved significantly (*P* < 0.05), whereas no statistically significant differences were observed in the physical health domain, mental health domain, or SAS scores between the pre- and post-intervention assessments (*P* > 0.05), as shown in Figure 5.

#### 4.4.2 Post-intervention scores of all outcome measures were compared between the two groups of family caregivers.

**FIGURE 6:**
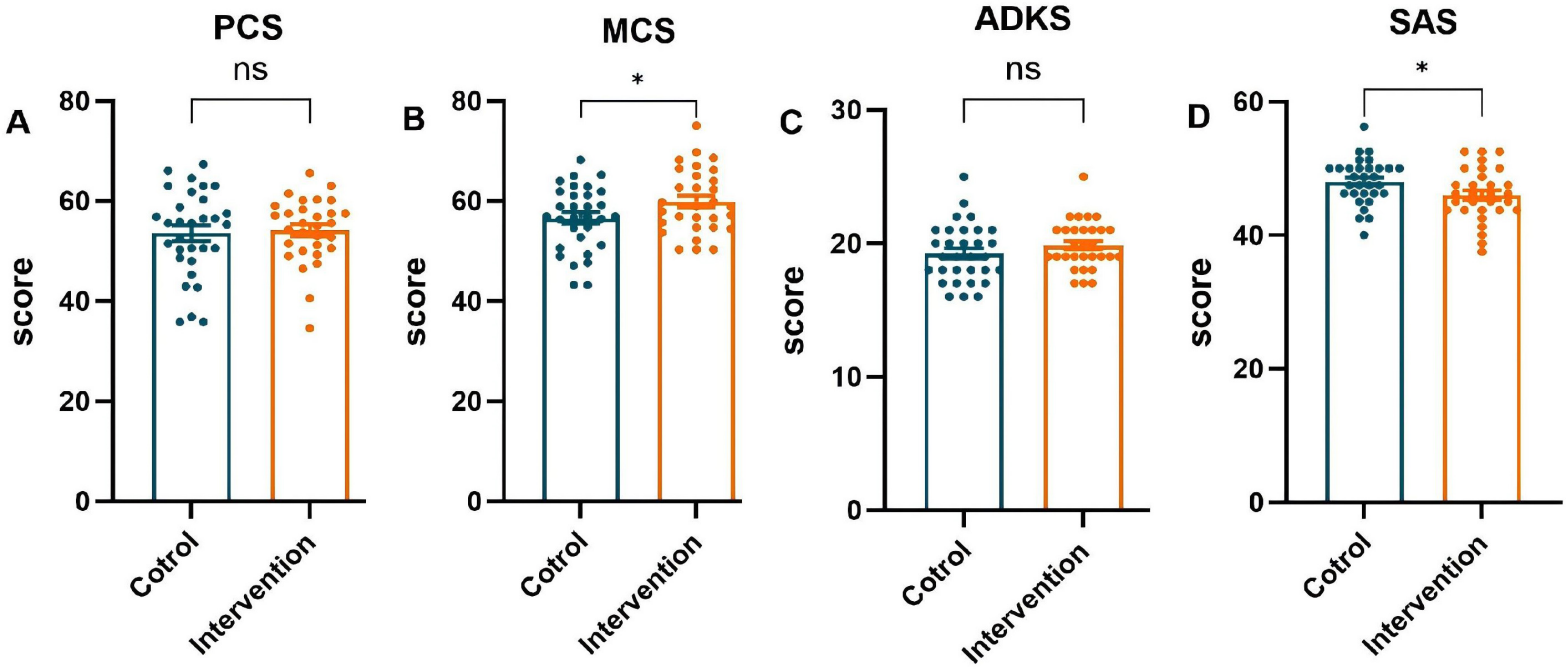
Post-intervention outcome scores: intervention group vs. control group. **(A)** The Physical Component Summary (PCS) score, **(B)** the Mental Component Summary (MCS) score, **(C)** the Chinese version of Alzheimer’s Disease Knowledge Scale (ADKS) score, **(D)** the Self-rating Anxiety Scale (SAS) score. *P < 0.05.

Post-intervention, AD caregivers in the intervention group reported significantly higher MCS scores (59.93 ± 6.68) and lower SAS scores (46.04 ± 3.91) than those in the control group (MCS: 56.20 ± 7.28; SAS: 48.06 ± 3.37) (both *P* < 0.05). In contrast, PCS and SDS scores did not differ meaningfully between the two groups (*P* > 0.05), as shown in Figure 6.

#### 4.4.3 quality of life scores by study stage: intervention group vs. control group patients

**FIGURE 7:**
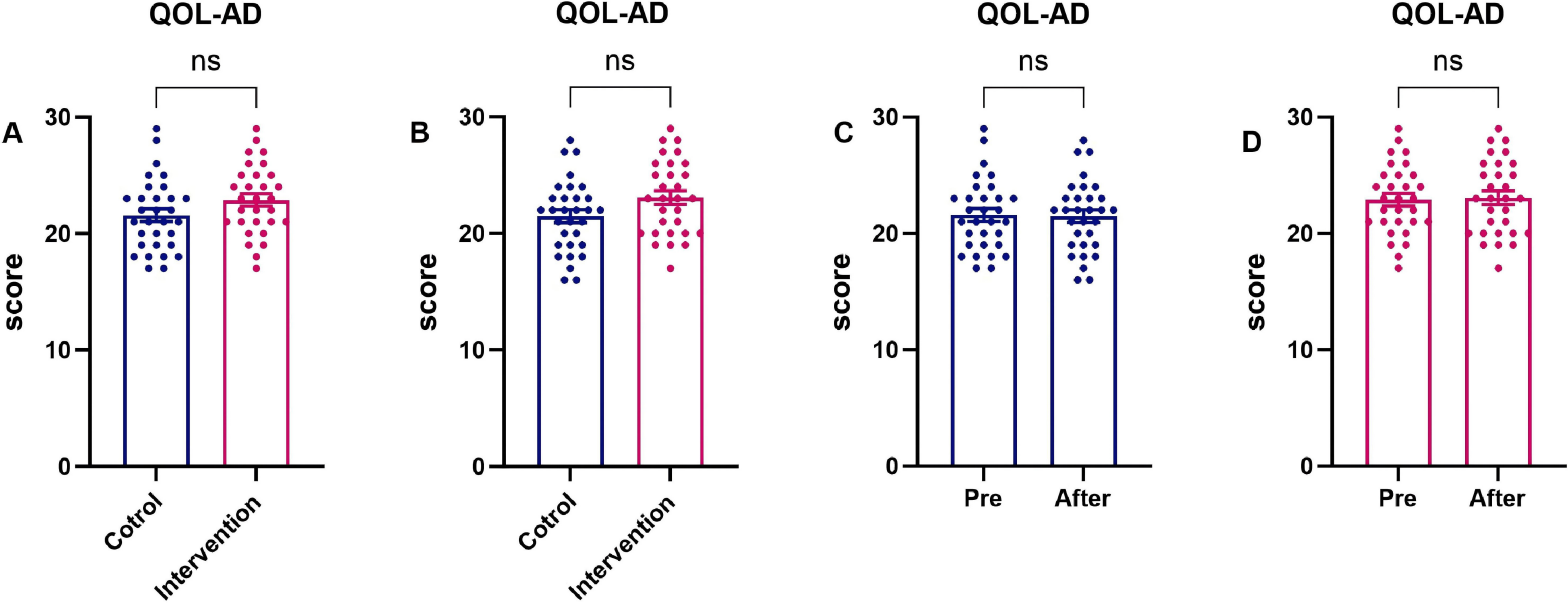
Quality of life scores by study stage: intervention group vs. control group patients. **(A)** Both groups at baseline (pre-Intervention); **(B)** both groups at post-intervention; **(C)** control group (pre-vs. post-Intervention); **(D)** intervention group (pre-vs. post-Intervention).

Quality of life did not differ significantly between the intervention and control groups, either at baseline or following the intervention (*P* > 0.05). This is shown in Figure 7, which details the scores by study stage.

## 5 Discussion

### 5.1 The effectiveness of the home care program for family caregivers of Alzheimer’s disease

The mean age of AD patients in this study was 75.49 ± 6.81 years. Of the 61 patients, 42 (68.85%) were female. Consistent with previous domestic and international studies (Han et al., 2020), AD prevalence was higher among individuals aged 75 years and older, with females exhibiting a greater prevalence than males. The mean age of the 61 family caregivers was 66.21 ± 11.94 years, with 33 (54.10%) being male. This predominance of male caregivers contrasts with data from international 2024 Alzheimer’s Association (2024). This discrepancy is potentially attributable to the high proportion of female patients in our cohort and the fact that 39 caregivers (63.93%) were spouses (who are typically male when the patient is female). This finding aligns with research by Chinese scholar Xu et al. (2022), who noted that spouses are often the primary AD family caregivers.

Among the caregivers, 22 were the patients’ children. Of these child caregivers, 16 (72.73%) were female, consistent with domestic and international research indicating that caregiving responsibilities among offspring frequently fall to daughters (Ohno et al., 2021; National Center for Neurological Disorders et al., 2024). The overall educational attainment among family caregivers was moderate, with the majority having completed primary school or higher. Although 7 caregivers (11.48%) reported no formal schooling, all demonstrated comprehension of and willingness to cooperate with the intervention program.

Pre-intervention analysis of general demographic data revealed no statistically significant differences (*P* > 0.05) between the patient and caregiver groups, ensuring baseline comparability and minimizing potential confounding effects on outcome measures.

### 5.2 Influence of the intervention program on the health-related quality of life of family caregivers

Health-related quality of life was the primary outcome measure. At baseline, the mean PCS score for caregivers was 54.52 ± 7.63. This was lower than reported by Ma et al. (2023), a discrepancy potentially attributable to the higher mean age of caregivers in our study (66.21) compared to Ma’s cohort (59.00). Furthermore, 65.57% (40/61) of our caregivers had at least one chronic condition. Qi et al. (2021) reported an even lower mean PCS score of 41.33 ± 9.71, likely related to their sample’s higher mean age (69.91 ± 9.93 years).

After three months, PCS scores in both groups showed a marginal decline compared to baseline, consistent with other research (González-Fraile et al., 2021). This suggests that information provision, training, and supportive interventions for informal caregivers may have minimal or no significant impact on caregiver physical HRQoL. However, other studies indicate that mixed-method effectiveness research in community settings can significantly enhance the physical health of dementia caregivers (Gaugler et al., 2024). In our intervention group, the difference between baseline and post-intervention PCS scores was not statistically significant. The observed gradual decline in caregiver physical health over time may stem from the fact that most AD patients in this study were in moderate to severe stages. At these stages, patients’ self-care abilities markedly diminish, increasing the physical burden on caregivers and potentially leading to progressive health deterioration (Kim et al., 2024). The complex relationship between various scale scores, perceived benefits, and actual physical health changes highlights the difficulty in measuring physical health improvements and may also reflect characteristics of our study sample.

The baseline mental component summary (MCS) score for caregivers in this study was 55.93 ± 6.61, which was higher than that reported in some prior studies (Qi et al., 2021; Ma et al., 2023). This difference may be associated with the fact that the majority of caregivers were spouses, who often perceive caregiving as a duty and responsibility, potentially fostering a more composed attitude. Conversely, when adult children are caregivers, intergenerational differences, lifestyle conflicts, and communication barriers due to patient cognitive decline can strain the caregiver-patient relationship. Research demonstrates that such strained relationships negatively impact caregiver mental health, increasing psychological stress and emotional distress among child caregivers (Gibbons et al., 2019; Kim et al., 2024). Crucially, our analysis revealed a significant between-group difference in mental health improvement. While MCS scores increased in both the intervention and control groups post-intervention, the improvement was significantly greater in the intervention group (as detailed in Figure 6).

The superior mental health outcomes in the intervention group can likely be attributed to the active ingredients of our program. The structured training in coping strategies may have equipped caregivers with more effective tools to manage emotional distress, reduce feelings of isolation, and reframe the caregiving experience. In contrast, caregivers in the control group, who received usual care and baseline assessments, lacked access to these targeted resources, which may explain their comparatively modest improvement. These results align with and extend previous research demonstrating the efficacy of structured interventions for caregiver mental health(Xiao et al., 2025). The key advancement highlighted here is the demonstration of a clear comparative advantage over usual care. It is important to note, however, that the generalizability of these findings may be influenced by sample characteristics, such as the predominance of spousal caregivers. Future studies should investigate whether similar benefits are observed among adult child caregivers or in different cultural contexts.

### 5.3 The intervention program improved family caregivers’ knowledge of Alzheimer’s disease

The Chinese government has progressively intensified its focus on Alzheimer’s disease. In 2020, the National Health Commission of China initiated a strategic plan to enhance professional services for AD prevention and treatment. The following year, 2021, saw the publication of the Proposal for China’s Strategic Action Plan for Alzheimer’s Disease, which underscored the necessity of bolstering public education and raising AD awareness. The Guidelines for Early Prevention of Alzheimer’s Disease in China (2024) further encourage community health centers, hospitals, and healthcare institutions to proactively conduct health education on early AD prevention and disseminate related knowledge. Additionally, the National Health Commission, in conjunction with 14 other governmental departments, jointly issued the National Action Plan for Dementia in the Elderly (2024–2030). This plan sets objectives to disseminate scientific knowledge on dementia prevention and control and enhance caregiving capabilities by 2030.

The Alzheimer’s Disease Knowledge Scale (ADKS) comprehensively assesses understanding of AD. In this study, the mean baseline ADKS score for family caregivers was 18.38 ± 1.98. This score is lower than that reported by Sun et al. (2024) in a cross-sectional study conducted in Zhuhai, an economically advanced region of China. This discrepancy may be partly explained by demographic differences: the average age of respondents in Sun Yajun et al.’s study was 43.5 years (younger than our participants), and over 85% had completed at least primary school education. In contrast, the majority of participants in our study resided in western China, where economic and cultural development is relatively lagging, and most had only completed primary or junior high school education. Wang et al. (2022) revealed that 72.4% of family caregivers lacked clarity regarding necessary AD care knowledge and skills. The study (Elbejjani et al., 2021) emphasized the critical need to enhance public AD awareness, particularly in low- and middle-income countries, where family members often serve as primary caregivers. Recent studies indicate that (Ma et al., 2022; Zuo et al., 2023) even in economically and medically advanced regions of eastern China, medical and community workers exhibit low levels of AD knowledge. While a disparity in knowledge exists between the general population and healthcare professionals, health education has been demonstrated to significantly improve short-term AD knowledge among the public (Perales et al., 2020).

Both conventional intervention methods and the program employed in this study effectively enhanced family caregivers’ ADKS scores. Statistically significant differences (*P* < 0.05) were observed between pre- and post-intervention ADKS scores.

### 5.4 The intervention program alleviates the anxiety of family caregivers

The baseline assessment revealed a high level of anxiety among AD family caregivers in this study, with an average Self-Rating Anxiety Scale (SAS) score of 47.73 ± 3.77. This score is notably higher than that reported for caregivers of patients with other conditions, such as lung cancer (Wang, 2023), underscoring the substantial emotional burden associated with dementia caregiving. This elevated anxiety likely stems from the progressive, unpredictable nature of Alzheimer’s disease, which demands long-term, multifaceted care and poses escalating challenges (Kokkosis and Tsirka, 2020; Wang et al., 2022).

Most importantly, the post-intervention analysis demonstrated a superior reduction in anxiety for the intervention group (*P* < 0.05) and an alleviation of the primary caregivers’ emotional burden—a finding consistent with prior research (Lappalainen et al., 2019). While both groups were assessed over time, caregivers who received our integrated intervention exhibited a significantly greater decrease in SAS scores compared to those in the care-as-usual control group (as detailed in Figure 6). This between-group difference is a pivotal finding, as it indicates that the anxiety reduction was not merely a function of time or assessment reactivity but was specifically attributable to the intervention components. The mechanisms for this reduction can be traced to the intervention’s components: educational sessions enhanced caregivers’ understanding of patient conditions and care requirements, thereby reducing knowledge-related anxiety, while professional skills training improved their proficiency in managing caregiving challenges, further decreasing anxiety levels. These findings align with existing evidence that counseling, psychoeducation, skill development, and multicomponent interventions targeting both patients and caregivers can improve caregiver well-being and delay patient institutionalization (Shi et al., 2020).

### 5.5 Effects of the intervention program on the quality of life of Alzheimer’s patients

The results indicated that while the control group’s Quality of Life (QoL) score exhibited a slight decline after 3 months, the intervention group demonstrated marginal improvement. However, neither group showed statistically significant differences between post-intervention and baseline measurements (*P* > 0.05). This contrasts with Zhang et al.’s findings Zhang et al.’s (2023), where regression family intervention significantly improved patient QoL. The discrepancy may stem from their patient cohort’s younger average age and superior baseline cognitive function.

Consistent with Chen et al. (2019), play therapy did not significantly enhance AD patients’ QoL. In our study, most participants had moderate-to-severe AD-likely because early symptoms (e.g., mild memory impairment) are frequently overlooked as normal aging (Zhang et al., 2021), delaying diagnosis until advanced stages when interventions show limited efficacy.

Although enhancing patient QoL is a therapeutic priority, disease progression typically diminishes memory, attention, and communication skills. This study used caregiver-rated QoL-AD scores, as caregiver proxies are generally reliable (Engel et al., 2024). However, caregiver assessments may introduce bias. During moderate-severe stages, patients experience significant memory loss, neuropsychiatric symptoms, and behavioral challenges that markedly reduce QoL. While the intervention didn’t significantly improve QoL scores, it prevented further deterioration.

### 5.6 Practical implications, cost-effectiveness, and societal value

Although effect sizes were modest, the intervention required minimal resources and demonstrated high feasibility. Structured caregiver education, once established, incurs low recurring costs relative to pharmacotherapy or institutional care—a decisive advantage in resource-constrained settings such as western China. For health systems and families facing financial strain, incremental gains in caregiver quality of life and care quality achieved with minimal investment constitute a clinically meaningful outcome.

The protocol’s broader value lies in promoting equitable access to dementia support. By engaging a low-education cohort-typically underserved-it offers a replicable model for vulnerable populations. Scaled implementation could empower families to defer institutionalization, yielding indirect savings for public payers. A formal cost-utility analysis and multicenter implementation trials are now warranted to quantify these savings and optimize scalability.

## 6 Limitations

This study evaluated the clinical application of the developed care protocol and verified its practical feasibility. The protocol holds significant value for guiding family caregivers in home-based Alzheimer’s disease care, as it enhances care quality, improves caregivers’ health-related quality of life, and strengthens their Alzheimer’s knowledge retention. However, there are some limitations in this study: (1) The sample size of this study was small, and participants were recruited exclusively from family caregivers of Alzheimer’s disease patients at a single tertiary hospital. This recruitment approach may have contributed to the high attrition rate and could limit the representativeness of the sample. The dropout rate (15.28%) was primarily attributable to external logistical factors, which is consistent with findings from prior caregiver intervention trials. Although a longer follow-up period might increase attrition, sensitivity analyzes confirmed the robustness of the primary results. Future studies could evaluate remote contact and incentive strategies to maintain participant retention beyond 3 months. Additionally, the enrolled AD patients had notably low levels of education, a characteristic representative of the older population in our catchment area (Zunyi, an economically underdeveloped region in western China). This limited educational range may restrict the external validity of the findings when generalized to settings with higher-literacy patients. Nevertheless, it also demonstrates the feasibility of the protocol among underserved, low-health-literacy populations-groups often excluded from clinical trials. (2) Reliance on subjective measures. Outcome assessment relied exclusively on scale scores, which are inherently subjective. Specifically, cognitive status was screened solely with the Montreal Cognitive Assessment (MoCA); although convenient for clinical practice, this brief tool lacks the depth of a comprehensive neuropsychological battery. Future studies should therefore supplement subjective ratings with objective biomarkers (e.g., actigraphy-derived sleep–wake parameters or salivary cortisol) and, whenever feasible, employ full neuropsychological evaluations to improve the validity and generalizability of the findings. (3) Study duration and follow-up, The intervention period was limited to 3 months, with a correspondingly short follow-up period. This constraint precluded a comprehensive assessment of the intervention’s long-term effects. Future studies should extend both the intervention and follow-up periods to better evaluate sustained.

## 7 Conclusion

Facing China’s aging crisis, most Alzheimer’s patients rely on family caregivers lacking professional support. This study developed a home-care intervention protocol and tested efficacy through a 3-month RCT with 61 AD families (intervention = 31, control = 30). This study had an overall attrition rate of 15.28%, primarily due to the poor health status of patients or family caregivers, which correlated with the perceived stress experienced by caregivers. The findings demonstrated that the program effectively enhanced the mental health status of family caregivers, increased their awareness of Alzheimer’s disease-related knowledge, and alleviated their anxiety levels. Although the quality of life in the intervention group did not show statistically significant improvement, there was an increase in the total QoL-AD score. These results suggest that the protocol employed in this study holds potential clinical significance.

## Data Availability

The raw data supporting the conclusions of this article will be made available by the authors, without undue reservation.

## References

[B1] Alzheimer’s Association (2024). 2024 Alzheimer’s disease facts and figures. *Alzheimers Dement* 20 3708–3821. 10.1002/alz.13809 38689398 PMC11095490

[B2] Balvert S. Del Sordo G. C. Milders M. V. (2024). The efficacy of dyadic interventions for community-dwelling people with dementia and their caregivers: a systematic review and meta-analysis. *Ageing Res. Rev.* 96:102258. 10.1016/j.arr.2024.102258 38479479

[B3] Carpenter B. D. Balsis S. Otilingam P. G. Hanson P. K. Gatz M. (2009). The Alzheimer’s Disease Knowledge Scale: development and psychometric properties. *Gerontologist* 49 236–247. 10.1093/geront/gnp023 19363018 PMC2667675

[B4] Chen Y. Dai M. Zhu H. Shen Q. (2019). Effects of play therapy on cognitive function, emotional state and quality of life in patients with Alzheimer’s disease: a meta-analysis. *Zhejiang Med. J.* 41 1383–1386. 10.12056/j.issn.1006-2785.2019.41.13.2018-2453

[B5] Cox C. G. Brush B. L. Kobayashi L. C. Roberts J. S. (2025). Determinants of dementia diagnosis in U.S. primary care in the past decade: a scoping review. *J. Prevent. Alzheimer’s Dis.* 12:100035. 10.1016/j.tjpad.2024.100035 39863322 PMC12184027

[B6] Dahlberg L. Demack S. Bambra C. (2007). Age and gender of informal carers: a population-based study in the UK. *Health Soc. Care Commun.* 15 439–445. 10.1111/j.1365-2524.2007.00702.x 17685989

[B7] Elbejjani M. Wahab K. El Hachem R. Tanielian M. Feghali L. Assaf G. (2021). Knowledge and attitude towards Alzheimer’s disease and related dementias in a low- to middle-income country: a cross-sectional survey among Lebanese middle-aged and older adults. *Psychogeriatrics* 21 699–708. 10.1111/psyg.12722 34107555

[B8] Engel L. Sokolova V. Bogatyreva E. Leuenberger A. (2024). Understanding the influence of different proxy perspectives in explaining the difference between self-rated and proxy-rated quality of life in people living with dementia: a systematic literature review and meta-analysis. *Qual. Life Res.* 33 2055–2066. 10.1007/s11136-024-03660-w 38656407 PMC11286712

[B9] Gaugler J. E. Baier R. R. Baker Z. G. Boltz M. Fortinsky R. H. Gustavson A. M. (2024). Using hybrid effectiveness studies to facilitate implementation in community-based settings: three case studies in dementia care research. *J. Am. Med. Dir. Assoc.* 25 27–33. 10.1016/j.jamda.2023.07.025 37643720 PMC10840611

[B10] Gibbons S. W. Ross A. Wehrlen L. Klagholz S. Bevans M. (2019). Enhancing the cancer caregiving experience: Building resilience through role adjustment and mutuality. *Eur. J. Oncol. Nurs.* 43:101663. 10.1016/j.ejon.2019.09.004 31606005 PMC6953477

[B11] González-Fraile E. Ballesteros J. Rueda J. R. Santos-Zorrozúa B. Solà I. McCleery J. (2021). Remotely delivered information, training and support for informal caregivers of people with dementia. *Cochrane Database Syst. Rev.* 1:CD006440. 10.1002/14651858.CD006440.pub3 33417236 PMC8094510

[B12] Han S. Ren Y. Ma X. (2020). Trend and prediction of disease burden of Alzheimer’s disease and other dementias in the elderly in China: a comprehensive analysis based on GBD 2021. *Chin. Journal of General Practice* 28 996–1003. 10.1007/s10620-025-09584-w 41307862

[B13] He R. Jing C. Li B. Pang G. Yu H. Sun L. (2013). Reliability and validity of the Chinese version of Alzheimer’s Disease Knowledge Scale. *Chin. J. Nurs.* 48 835–837. 10.3761/j.issn.0254-1769.2013.09.022

[B14] Homma A. Meguro K. Dominguez J. Sahadevan S. Wang Y. H. Morris J. C. (2006). Clinical dementia rating workshop: the Asian experience. *Alzheimer Dis. Assoc. Disord.* 20 318–321. 10.1097/01.wad.0000213869.32676.d8 17132982

[B15] Huang Y. (2015). Random error control and sample size determination in medical research. *Chin. J. Ment. Health* 11 874–880.

[B16] Kim H. Engström G. Sadak T. Emami A. (2024). Characteristics and correlates of perceived physical and psychological health among family caregivers living with persons with advanced dementia. *West J. Nurs. Res.* 46 104–113. 10.1177/01939459231217923 38112102

[B17] Kojima Y. Yamada S. Kamijima K. Kogushi K. Ikeda S. (2024). Burden in caregivers of patients with schizophrenia, depression, dementia, and stroke in Japan: comparative analysis of quality of life, work productivity, and qualitative caregiving burden. *BMC Psychiatry* 24:591. 10.1186/s12888-024-06000-x 39223532 PMC11370303

[B18] Kokkosis A. G. Tsirka S. E. (2020). Neuroimmune mechanisms and sex/gender-dependent effects in the pathophysiology of mental disorders. *J. Pharmacol. Exp. Therapeut.* 375 175–192. 10.1124/jpet.120.266163 32661057 PMC7569311

[B19] Kor P. Liu J. Chien W. T. (2019). Effects of a modified mindfulness-based cognitive therapy for family caregivers of people with dementia: A pilot randomized controlled trial. *Int. J. Nurs. Stud.* 98 107–117. 10.1016/j.ijnurstu.2019.02.020 30922609

[B20] Lappalainen P. Pakkala I. Nikander R. (2019). CareACT - internet-based intervention for enhancing the psychological well-being of elderly caregivers - a study protocol of a controlled trial. *BMC Geriatr.* 19:72. 10.1186/s12877-019-1071-9 30836951 PMC6402103

[B21] Li, Lu W. H. Shen Y. (2002). The effectiveness of a community nurse-led support program for dementia caregivers in chinese communities: the Chongqing ageing and dementia study. *Chin. J. Prevent. Med.* 36 109–113. 10.3760/j:issn:0253-9624.2002.02.011 38025803 PMC10657713

[B22] Liang C. S. Li D. J. Yang F. C. Tseng P. T. Carvalho A. F. Stubbs B. (2021). Mortality rates in Alzheimer’s disease and non-Alzheimer’s dementias: a systematic review and meta-analysis. *Lancet. Healthy Longevity* 2 e479–e488. 10.1016/S2666-7568(21)00140-9 36097997

[B23] Livingston G. Huntley J. Sommerlad A. Ames D. Ballard C. Banerjee S. (2020). Dementia prevention, intervention, and care: 2020 report of the Lancet Commission. *Lancet* 396 413–446. 10.1016/S0140-6736(20)30367-6 32738937 PMC7392084

[B24] Logsdon R. G. Gibbons L. E. McCurry S. M. Teri L. (2002). Assessing quality of life in older adults with cognitive impairment. *Psychosom. Med.* 64 510–519. 10.1097/00006842-200205000-00016 12021425

[B25] Ma W. Zhu L. Tang J. Diao W. Qian L. Feng X. (2022). Testing the knowledge of Alzheimer’s disease via an intervention study among community health service center staff in Jiaxing, China. *Front. Public Health* 10:969653. 10.3389/fpubh.2022.969653 36777777 PMC9911520

[B26] Ma Y. Gong J. Zeng L. Wang Q. Yao X. Li H. (2023). The effectiveness of a community nurse-led support program for dementia caregivers in chinese communities: the Chongqing ageing and dementia study. *J. Alzheimer’s Dis. Rep.* 7 1153–1164. 10.3233/ADR-230067 38025803 PMC10657713

[B27] Matthews K. A. Xu W. Gaglioti A. H. Holt J. B. Croft J. B. Mack D. (2019). Racial and ethnic estimates of Alzheimer’s disease and related dementias in the United States (2015-2060) in adults aged ≥65 years. *Alzheimer’s Dement.* 15 17–24. 10.1016/j.jalz.2018.06.3063 30243772 PMC6333531

[B28] Mattos M. K. Bernacchi V. Shaffer K. M. Gallagher V. Seo S. Jepson L. (2024). Sleep and caregiver burden among caregivers of persons living with dementia: a scoping review. *Innov. Aging* 8:igae005. 10.1093/geroni/igae005 38420182 PMC10901478

[B29] Nasreddine Z. S. Phillips N. A. Bédirian V. Charbonneau S. Whitehead V. Collin I. (2005). The montreal cognitive assessment, MoCA: a brief screening tool for mild cognitive impairment. *J. Am. Geriatr. Soc.* 53 695–699. 10.1111/j.1532-5415.2005.53221.x 15817019

[B30] National Center for Neurological Disorders, Xuanwu Hospital, Capital Medical University, National Center for Chronic and Noncommunicable Disease Control and Prevention, Chinese Center for Disease Control and Prevention, and National Health Commission Capacity Building and Continuing Education Center. (2024). Blue paper on Alzheimer′s disease in China(simplified version). *Chin. Med. J.* 104 2701–2727. 10.3760/cma.j.cn12137-20240416-00883 38783696

[B31] Ohno S. Chen Y. Sakamaki H. Matsumaru N. Yoshino M. Tsukamoto K. (2021). Burden of caring for Alzheimer’s disease or dementia patients in Japan, the US, and EU: results from the National Health and Wellness Survey: a cross-sectional survey. *J. Med. Econ.* 24 266–278. 10.1080/13696998.2021.1880801 33538195

[B32] Perales J. Moore W. T. Fernandez C. Chavez D. Ramirez M. Johnson D. (2020). Feasibility of an Alzheimer’s disease knowledge intervention in the Latino community. *Ethn. Health* 25 747–758. 10.1080/13557858.2018.1439899 29457466 PMC6098744

[B33] Qi J. Guo Q. Yang Q. Zhang S. (2021). The mediating effect of self-efficacy of caregivers of dementia patients between patient-related impairment and health-related quality of life of caregivers. *Chin. J. Modern Nurs.* 21:641. 10.1186/s12877-021-02595-y 34772361 PMC8588578

[B34] Rosin E. R. Blasco D. Pilozzi A. R. Yang L. H. Huang X. (2020). A narrative review of Alzheimer’s Disease Stigma. *J. Alzheimer’s Dis.* 78 515–528. 10.3233/JAD-200932 33044185 PMC7739963

[B35] Ruggiano N. Brown E. L. Clarke P. J. Hristidis V. Roberts L. Framil Suarez C. V. (2024). An evidence-based IT program with chatbot to support caregiving and clinical care for people with dementia: the careheroes development and usability pilot. *JMIR Aging* 7:e57308. 10.2196/57308 39727199 PMC11684532

[B36] Shi H. Mao C. Tang J. Liang H. (2020). Research on the health of and interventions for family caregivers of people with dementia: a bibliometric analysis of research output during 1988-2018. *BMC Geriatr.* 20:20. 10.1186/s12877-020-1421-7 31964344 PMC6975077

[B37] Sun Y. J. Song J. Li X. P. Wang X. H. Wu Y. X. Huang J. J. (2024). Knowledge of Alzheimer’s disease and associated factors among adults in Zhuhai, China: a cross-sectional analysis. *BMC Public Health* 24:1769. 10.1186/s12889-024-19289-w 38961390 PMC11220978

[B38] Sun Y. Ji M. Leng M. Li X. Zhang X. Wang Z. (2022). Comparative efficacy of 11 non-pharmacological interventions on depression, anxiety, quality of life, and caregiver burden for informal caregivers of people with dementia: a systematic review and network meta-analysis. *Int. J. Nurs. Stud.* 129:104204. 10.1016/j.ijnurstu.2022.104204 35247788

[B39] Tian J. Xie H. Wang L. (2021). Chinese guidelines for the diagnosis and treatment of Alzheimer’s disease and dementia (2020 edition). *Chin. J. Geriatr.* 40 269–283. 10.3760/cma.j.issn.0254-9026.2021.03.001

[B40] Wang G. Qi J. Liu X. Ren R. Lin S. Hu Y. (2024). Chinese Alzheimer’s disease report 2024. *Diagnost. Theory Pract.* 23 219–256. 10.16150/j.1671-2870.2024.03.001

[B41] Wang H. (2023). *Current Status and Influencing Factors of Anxiety and Depression in Main Caregivers of Patients with Lung Cancer Surgery.* Hefei: Anhui Medical University, 10.26921/d.cnki.ganyu.2023.002258

[B42] Wang L. Wu S. Shi Y. Li R. Qun L. U. (2022). Correlation among anxiety, depression and social support of family caregivers of elderly patients with dementia at home. *Chin. J. Modern Nurs.* 28 2289–2295. 10.3760/cma.j.cn115682-20210929-04445

[B43] Wang W. Wang L. N. (2007). Application of the Montreal Cognitive Assessment in screening patients with mild cognitive impairment. *Chin. J. Internal Med.* 46 414–416.

[B44] Wang X. Chen H. Wang Y. Zheng D. Li J. (2024). Reliability and validity of Zung self-rating Anxiety Scale in community residents. *J. Clin. Psychiatry* 34 397–401. 10.3969/j.issn.1005-3220.2024.05.016

[B45] Williams K. Blyler D. Vidoni E. D. Shaw C. Wurth J. Seabold D. (2018). A randomized trial using telehealth technology to link caregivers with dementia care experts for in-home caregiving support: FamTechCare protocol. *Res. Nurs. Health* 41 219–227. 10.1002/nur.21869 29504666 PMC6003850

[B46] Xiao L. Ullah S. Hu R. Wang J. Wang H. Chang C. C. (2025). Corrigendum to “The effects of a facilitator-enabled online multicomponent iSupport for dementia programme: a multicentre randomised controlled trial”[Int. J. Nurs. Stud. 159 (2024) 104868]. *Int. J. Nurs. Stud.* 162:104960. 10.1016/j.ijnurstu.2024.104960 39572318

[B47] Xu Y. Wang J. Wang H. Li K. Wang H. Wang X. (2022). Status of awareness and demand for Alzheimer’s disease in China in 2022. *Alzheimer’s Dis. Relat. Dis.* 5 265–277. 10.3969/j.issn.2096-5516.2022.04.002

[B48] Yang J. Shi Q. Li P. Yu S. Wang Y. Chen X. (2022). Evaluation of reliability and validity of Chinese version of SF-36 scale in patients with chronic Keshan disease. *Chin. J. Endemiol.* 41 27–31. 10.3760/cma.j.cn231583-20201013-00262

[B49] Yang S. Zhang Y. Xie S. Chen Y. Jiang D. Luo Y. (2020). Predictors of perceived social support for patients with dementia: a mixed-methods study. *Clin. Interv. Aging* 15 595–607. 10.2147/CIA.S249223 32431493 PMC7201008

[B50] Yang Y. W. Hsu K. C. Wei C. Y. Tzeng R. C. Chiu P. Y. (2021). Operational determination of subjective cognitive decline, mild cognitive impairment, and dementia using sum of boxes of the clinical dementia rating scale. *Front. Aging Neurosci.* 13:705782. 10.3389/fnagi.2021.705782 34557083 PMC8455062

[B51] Zhang H. Ai Y. Wu Y. He R. Gao J. Wang X. (2013). Reliability and validity of the Chinese version of Quality of Life-Alzheimer’s Disease (QOL-AD). *Chin. J. Health Stat.* 30 57–59. 10.1186/s12887-025-05649-x 40217189 PMC11987452

[B52] Zhang R. Zhu J. Hu J. Zhang L. Dong C. (2023). Effects of return to family intervention on cognitive function and quality of life in patients with Alzheimer’s disease. *Chin. J. Nurs.* 38 106–110. 10.3870/j.issn.1001-4152.2023.22.106

[B53] Zhang X. X. Tian Y. Wang Z. T. Ma Y. H. Tan L. Yu J. T. (2021). The epidemiology of Alzheimer’s disease modifiable risk factors and prevention. *J. Prevent. Alzheimer’s Dis.* 8 313–321. 10.14283/jpad.2021.15 34101789 PMC12280729

[B54] Zung W. W. (1971). A rating instrument for anxiety disorders. *Psychosomatics* 12 371–379. 10.1016/S0033-3182(71)71479-0 5172928

[B55] Zuo S. Wang Y. Wang Z. Chen S. Liang J. Meng H. (2023). Alzheimer’s disease knowledge of nursing staff in East China: a latent profile analysis. *Nurs. Open* 10 6972–6979. 10.1002/nop2.1952 37483069 PMC10495715

